# Antibiotic-Loaded Bioglass 45S5 for the Treatment and Prevention of *Staphylococcus aureus* Infections in Orthopaedic Surgery: A Novel Strategy Against Antimicrobial Resistance

**DOI:** 10.3390/pathogens14080760

**Published:** 2025-08-01

**Authors:** Humera Sarwar, Richard A. Martin, Heather M. Coleman, Aaron Courtenay, Deborah Lowry

**Affiliations:** 1 School of Pharmacy and Pharmaceutical Sciences, Ulster University, Cr Road, Coleraine BT52 1SA, UK; sarwar-h1@ulster.ac.uk (H.S.); a.courtenay@ulster.ac.uk (A.C.); 2 College of Engineering and Physical Sciences, Aston University, Birmingham B4 7ET, UK; r.a.martin@aston.ac.uk

**Keywords:** Bioglass 45S5, antibiotic delivery, orthopaedic infections, *Staphylococcus aureus*, antimicrobial resistance, osteogenic potential

## Abstract

This study explores the potential of biodegradable Bioglass 45S5 formulations as a dual-function approach for preventing and treating *Staphylococcus aureus* infections in orthopaedic surgery while addressing the growing concern of antimicrobial resistance (AMR). The research focuses on the development and characterisation of antibiotic-loaded BG45S5 formulations, assessing parameters such as drug loading efficiency, release kinetics, antimicrobial efficacy, and dissolution behaviour. Key findings indicate that the F2l-BG45S5-T-T-1.5 and F2l-BG45S5-T-V-1.5 formulations demonstrated controlled antibiotic release for up to seven days, with size distributions of D(10): 7.11 ± 0.806 µm, 4.96 ± 0.007 µm; D(50): 25.34 ± 1.730 µm, 25.20.7 ± 0.425 µm; and D(90): 53.7 ± 7.95 µm, 56.10 ± 0.579 µm, respectively. These formulations facilitated hydroxyapatite formation on their surfaces, indicative of osteogenic potential. The antimicrobial assessments revealed zones of inhibition against methicillin-susceptible *Staphylococcus aureus* (MSSA, ATCC-6538) measuring 20.3 ± 1.44 mm and 24.6 ± 1.32 mm, while for methicillin-resistant *Staphylococcus aureus* (MRSA, ATCC-43300), the inhibition zones were 21.6 ± 1.89 mm and 22 ± 0.28 mm, respectively. Time-kill assay results showed complete bacterial eradication within eight hours. Additionally, biocompatibility testing via MTT assay confirmed cell viability of >75%. In conclusion, these findings highlight the promise of antibiotic-loaded BG45S5 as a multifunctional biomaterial capable of both combating bone infections and supporting bone regeneration. These promising results suggest that in vivo studies should be undertaken to expedite these materials into clinical applications.

## 1. Introduction

Orthopaedic implant-associated bone infections, including osteomyelitis and periprosthetic joint infections, remain among the most devastating complications in musculoskeletal surgery. Osteomyelitis can arise from open fractures, contiguous spread, or haematogenous seeding and is characterised by progressive inflammation, bone necrosis, and sequestra formation, often leading to chronic disease if not eradicated [1,2,3]. *Staphylococcus aureus* (SA), particularly methicillin-resistant strains (MRSA), are the most frequent pathogen, owing to their property for biofilm formation on both living bone and implant surfaces [4,5,6].

Current standard of care involves extensive surgical debridement combined with prolonged systemic antibiotic regimens. While systemic therapy can achieve bactericidal serum levels, its penetration into avascular bone sequestra or biofilm is limited, and prolonged high dose courses carry risks of nephrotoxicity, ototoxicity, and antibiotic resistance selection elsewhere in the body [3,7]. Local antibiotic delivery via non-degradable polymethylmethacrylate (PMMA) beads or calcium phosphate cements provides higher periprosthetic concentrations, but PMMA requires secondary removal surgery and exhibits suboptimal elution kinetics (burst release followed by rapid decline), while some calcium phosphate carriers provoke inflammatory responses as they degrade [8,9,10].

Bioglass^®^ 45S5 (45 wt% SiO_2_, 24.5 wt% Na_2_O, 24.5 wt% CaO, 6 wt% P_2_O_5_) was pioneered by Hench et al. and remains the archetype of bioactive glasses [11]. Upon implantation, it undergoes surface reactions that result in the rapid formation of a hydroxycarbonate apatite layer, chemically and structurally akin to bone mineral, thereby promoting osteoblast adhesion, proliferation, and new bone formation [12,13]. Simultaneously, ionic dissolution products (e.g., Si (OH)_4_, Ca^2+^, Na^+^, PO_4_^3−^) can elevate local pH and osmolarity, exerting an inherent antimicrobial effect against both planktonic bacteria and early biofilm formation [14]. When antibiotics are incorporated into this matrix, a synergistic effect is achieved: the intrinsic bactericidal properties of ion release, combined with controlled local delivery of antibiotics, provide a robust defense against SA infections [12,15].

The intrinsic antibacterial action of Bioglass 45S5 (BG45S5) is multifactorial. The ion release leads to an elevation in local pH and osmolarity, which in turn disrupts the bacterial cell membrane potential and interferes with essential cellular functions. These physicochemical changes create an environment in which bacteria such as SA are unable to adhere effectively or produce robust biofilms [16]. When antibiotics, for example, vancomycin, tetracycline, or gentamicin, are loaded into the Bioglass matrix, the resultant hybrid material exerts a dual-action antimicrobial attack: the sustained high local antibiotic concentrations complement the glass-induced bactericidal effects, delivering potent therapy against both planktonic bacterial populations and embedded biofilms [15]. The degradation process of BG45S5 is highly relevant to its performance as a local drug delivery system. As the glass matrix dissolves, antibiotics encapsulated within or chemically bound to the surface are liberated in a controlled manner. Release kinetics depend upon parameters such as pore size, degree of interconnected porosity, and the strength of the electrostatic interactions between the antibiotic and the glass surface [17].

In vitro studies demonstrate that antibiotic-loaded BG45S5 substantially reduces the colony-forming units of SA within a short period and effectively disrupts established biofilms. These observations are particularly pertinent given that biofilm-associated bacteria exhibit up to a thousand-fold increase in resistance to systemic antibiotics compared to their planktonic counterparts [17,18]. The localised delivery system ensures that therapeutic antibiotic levels are sustained at the site of infection, thereby overcoming the diffusion barriers imposed by biofilms and reducing the risk of treatment failure [19].

In this study, we evaluate BG45S5 microparticles coated/primed with trehalose as crosslinker and loaded with vancomycin and teicoplanin for their physicochemical properties, antibiotic release kinetics, and efficacy against SA and MRSA. By combining potent localised antimicrobial delivery with controlled adsorption of antibiotic through a carbohydrate crosslinker with the osteogenic capacity of Bioglass, this approach aims to overcome current limitations in treating and preventing implant-associated bone infections and to contribute a novel weapon in the fight against antimicrobial resistance by using the physicochemical properties, antibiotic release kinetics, and efficacy against SA and MRSA. Vancomycin and teicoplanin were selected because glycopeptides remain the gold-standard agents for managing orthopaedic infections caused by SA, including MRSA. Both drugs inhibit late-stage peptidoglycan cross-linking, retain activity in the low-pH, high-ion milieu of infected bone, and display minimal cross-resistance with β-lactams [20]. Vancomycin is the first-line systemic therapy for acute implant-associated osteomyelitis and is routinely mixed into polymethyl-methacrylate spacers or calcium-sulfate beads to obtain local concentrations ≥1000 µg mL^−1^, levels that exceed serum trough values by two orders of magnitude yet remain safe because the drug is poorly absorbed systemically from bone cavities [21,22]. Teicoplanin offers two complementary advantages: a higher intrinsic potency against staphylococci (MIC_90_ 0.5–1 µg mL^−1^ versus 1–2 µg mL^−1^ for vancomycin) and an exceptionally long elimination half-life (40–70 h), which allows once-daily systemic dosing and sustains tissue levels in poorly perfused bone [23,24]. In vitro teicoplanin adsorbs strongly to hydroxyapatite and shows slower desorption than vancomycin providing a prolonged local reservoir without compromising osteoblast viability [25]. Employing both glycopeptides therefore enables a direct comparison between a fast-releasing, amphiphilic molecule and a more hydrophobic, slow-eluting analogue while covering the full susceptibility range of SA encountered in orthopaedic surgery. The aim of this work is to combine potent localised antimicrobial delivery with controlled adsorption of antibiotics through a carbohydrate crosslinker with the osteogenic capacity of Bioglass. This approach aims to overcome current limitations in treating and preventing implant-associated bone infections and to contribute a novel weapon in the fight against antimicrobial resistance.

## 2. Materials and Methods

### 2.1. Materials

Vancomycin HCl (500 mg powder for concentrate for solutions or infusions) was procured from Bowmed Ibisqus limited. Teicoplanin (Targocid^®^ 200 mg powder for solution for injection/infusion or oral solution) was procured from Sanofi. Phosphate-buffered saline (Dulbecco A) pH 7.4 and D9777-dialysis tubing cellular membrane were purchased from Sigma Aldrich. Additional materials were purchased as follows: Minisart Syringe Filter (sterile) 0.2 and 0.4 µm and Acetone for HPLC ≥ 99.8% (Sigma Aldrich, St. Louis, MO, USA), Acetonitrile for HPLC ≥ 99.9% (Sigma Aldrich, St. Louis, MO, USA), Methanol for HPLC ≥ 99.9% (Sigma Aldrich, St. Louis, MO, USA), Orthophosphoric acid 85% *w*/*w* (VWR International), Tryptic Soy Agar (VWR International, Radnor, PA, USA), Tryptic Soy Broth and Sterile loops (VWR International, Radnor, PA, USA), sterile L-shape spreader (VWR international, Radnor, PA, USA), SA (ATCC 6538) (Thermofischer Scientific, Waltham, MA, USA), MRSA (ATCC 43300) (from the frozen samples from microbiology lab), Gibco™ Dulbecco’s Modified Eagle Medium (DMEM) (Thermofischer Scientific, Waltham, MA, USA), Gibco™ penicillin/streptomycin (10,000 U/mL) (Thermofischer Scientific, Waltham, MA, USA), and Gibco™ Fetal bovine serum (Thermofischer Scientific, Waltham, MA, USA). Simulated body fluid was prepared by dissolving the chemical reagents according to the ISO 23317:2014 standard, i.e., NaCl, NaHCO_3_, KCl, K_2_HPO_4_ 3H_2_O, MgCl_2_ 6H_2_O, Na_2_SO_4,_ and CaCl_2_ in deionised water and buffering with tris(hydroxymethyl) aminomethane and 1 M HCl to obtain pH value of 7.42.

### 2.2. Methods

#### 2.2.1. Preparation of BG45S5

BG45S5 cores were synthesised via traditional melt-quenching, yielding dense, non-porous particles without internal pore networks. Precursors, consisting of H_6_NO_4_P, Na_2_CO_3_, CaCO_3_, and SiO_2_, were weighed to give the appropriate molar composition (SiO_2_)_46.1_ (CaO)_26.9_ (Na_2_O)_24.4_ (P2O5)_2.6_. Precursors were thoroughly mixed, placed in a 90% platinum/10% rhodium crucible (GLC alloys Ltd., Middlesex, UK) and then heated using an electric furnace. The precursors were heated from room temperature at a rate of 10 °C min^−1^ to 1450 °C and held at this temperature for 90 min. The molten liquid was poured into an open graphite at room temperature and allowed to cool. The glass was then ground with planetary ball mill (PM100, Retsch, Derbyshire, UK) and sieved to obtain the desired particle size distribution. The amorphous nature of the Bioglass was confirmed using XRD. Glasses were kept in a desiccator to prevent absorption of atmospheric moisture.

#### 2.2.2. Formulation Preparation Using Trehalose as Crosslinker with BG45S5

BG45S5 was coated with different concentrations of trehalose and 24 formulations were prepared which are listed in Appendix A (Table A1). BG45S5 was coated with trehalose dihydrate (MW = 378.33 g mol^−1^) in the weight ratios from 1:1 to 1:3. Each blend was dispersed in 15 mL deionised water in a 20 mL glass vial and magnetically stirred for 2 h at room temperature. The suspension was centrifuged at 8000 rpm for 10 min; the supernatant was collected for later analyses. Pellets were washed once with 5 mL phosphate-buffered saline (pH 7.4) and centrifuged again under identical conditions. The recovered microparticles were pre-frozen at –16 °C for 30 min and lyophilised for 24 h. After freeze-drying, samples were weighed to calculate mass loss and stored in airtight vials.

All formulations underwent iterative optimisation of particle size, zeta potential, and initial drug loading; only the data for the final, optimised formulation with the ratio of Bioglass/trehalose (1:3) codes as F2l-BG45S5-T are presented in this paper.

#### 2.2.3. Drug Loading in Blank Formulations

The pre-optimised Bioglass formulation—F2l-BG45S5-T—was weighed (500 mg) into separate glass beakers. Sterile vancomycin HCl and teicoplanin solutions were freshly prepared in deionised water at 0.5, 1.0, and 1.5% *w*/*v* and filtered (0.20 µm), and 10 mL of the desired solution was added to each beaker. Suspensions were magnetically stirred for 1 h at 25 °C, then transferred to 15 mL tubes and centrifuged (8000 rpm, 10 min). The supernatant was saved (4 °C) for later determination of loading efficiency. Pellets were washed once with 5 mL phosphate-buffered saline (pH 7.4) and re-centrifuged to remove loosely bound drug.

The resulting drug-loaded powders were pre-frozen (−16 °C, 30 min) and lyophilised for 24 h, after which the dry mass was recorded to calculate drug uptake (Equations (1)–(3)). Final samples were stored in airtight vials at 4 °C. Formulation codes after drug loading are listed in Table 1.

(1) $$D_{T}=D_{S1}+D_{S2}$$

(2) $$D_{L}=D_{sol}-D_{T}$$

(3) $$D\;(\%)=\frac{D_{L}}{D_{sol}}\times 100$$

where

*D_T_* = Total amount of drug in supernatant.
*D*_*S*1_ = Amount of drug in supernatant 1.
*D*_*S*2_ = Amount of drug in supernatant 2.
*D_L_* = Total amount of drug loaded.
*D_sol_* = Drug dissolved in the loading solution.
*D* (%) = Drug loading efficiency.

**Table 1:** Formulation codes after drug loading.

Drugs	Concentrations	Blank Formulations	Drug-Loaded Formulations
Vancomycin HCl	0.5% (*w*/*v*)	F2l-BG45S5-T	F2l-BG45S5-T-V-0.5
	1% (*w*/*v*)		F2l-BG45S5-T-V-1
	1.5% (*w*/*v*)		F2l-BG45S5-T-V-1.5
Teicoplanin	0.5% (*w*/*v*)	F2l-BG45S5-T	F2l-BG45S5-T-T-0.5
	1% (*w*/*v*)		F2l-BG45S5-T-T-1
	1.5% (*w*/*v*)		F2l-BG45S5-T-T-1.5

#### 2.2.4. Particle Size Analysis of the Loaded BG45S5 Formulations

BG45S5 formulations listed in Table 1 (~30 mg) were dispersed in 5 mL deionised water (6 mg mL^−1^) and introduced into a water-filled Hydro LV cell on a Mastersizer 3000 (Malvern). Measurements used the Mie model with the following optical parameters: particle refractive index (RI) = 1.514, dispersant RI = 1.330, absorption = 0.10. Data were collected at 1–30% laser obscuration and only results that met the instrument’s quality criteria were retained for calculation of volume-weighted size distributions and specific surface area.

#### 2.2.5. Zeta Potential (ZP) Analysis of the Blank and Drug-Loaded BG45S5 Formulations

Blank and antibiotic-loaded Bioglass powders (10 mg) were dispersed in 1 mL de-ionised water and analysed at 25 °C on a Zetasizer Nano (Malvern Panalytical) fitted with a dip-cell electrode. Electrophoretic mobility was converted to zeta potential (ζ) with the instrument software using the Smoluchowski approximation. Each formulation was measured in triplicate; results are reported as mean ± SD and RSD % to verify reproducibility.

#### 2.2.6. Scanning Electron Microscopy (SEM)

Surface morphology was examined on a TM3000 benchtop scanning electron microscope (Hitachi, Tokyo, Japan). Formulations in powders were mounted on aluminium stubs with conductive carbon tape and imaged in COMPO mode at 15 kV. Representative micrographs were collected at ×1.5 k, and ×3 k to visualise particle shape, surface roughness, and trehalose coating on blank and vancomycin- or teicoplanin-loaded BG45S5 (0.5–1.5% *w*/*v*). Images were recorded digitally and processed with instrument software for basic measurements and contrast enhancement.

#### 2.2.7. In Vitro pH Analysis of the Drug-Loaded Formulations in SBF

Triplicate samples (10 mg) of each formulation listed in Table 1 and of the blank BG45S5 cores were placed in sterile 20 mL scintillation vials. Sterile SBF (10 mL; pH 7.36) was added through 0.20 µm PES syringe filters (Minisart^®^ syringe filter, Sartorius Stedim Biotech GmbH, Göttingen, Germany). Vials were sealed and incubated at 37 °C on an orbital shaker (50 rpm). The medium pH was recorded with a Jenway 3510 m every 24 h for 14 days.

#### 2.2.8. In Vitro Drug Release Profiling of Antibiotic-Loaded Bioglass Microparticles

Drug-loaded Bioglass microparticles containing vancomycin HCl or teicoplanin at 0.5, 1.0, or 1.5% *w*/*w* (25 mg per test) were sealed in hydrated cellulose dialysis tubing (MWCO ≈ 14 kDa; 25 mm flat width, ~5 cm length). Each pouch was suspended in 10 mL phosphate-buffered saline (PBS, pH 7.4) and incubated at 37 °C with gentle agitation (50 rpm). At predefined time points from 15 min to 168 h, 1 mL of medium was withdrawn and immediately replaced with fresh PBS to maintain sink conditions. Drug concentrations in all samples were determined by HPLC (Table 2), and cumulative release profiles were plotted for each formulation.

#### 2.2.9. Antimicrobial Assay

##### Bacterial Strains

*Methicillin-susceptible staphylococcus aureus* (MSSA) ATCC 6538 and *methicillin-resistant S. aureus* (MRSA) ATCC 43,300 were used throughout. Each was streaked onto tryptone soy agar (TSA) and incubated 24 h at 37 °C to give master plates (stored 4 °C, ≤2 months). Fresh working plates were generated every two weeks.

##### Culture Growth

Three to five colonies from a working plate were transferred into 5 mL tryptone soy broth (TSB) and incubated overnight (18–24 h, 37 °C, 150 rpm).

##### Molecular Confirmation (16 S rDNA PCR)

DNA templates were prepared either by (i) colony PCR (colony boiled in 50 µL nuclease-free H_2_O, supernatant used directly) or (ii) Wizard^®^ genomic extraction (Promega). A 50 µL PCR contained 5 µL 10× buffer, 1.5 µL 50 mM MgCl_2_, 1 µL 10 mM dNTPs, 2.5 µL each of primers 9 bfm/1512 uR (10 µM), 0.5 µL Taq polymerase (5 U µL^−1^), 1 µL template and water to volume. Cycling: 95 °C × 3 min; 30 × (95 °C 30 s, 55 °C 30 s, 72 °C 90 s); 72 °C × 5 min. A single ~1.5 kb band on 1% agarose confirmed strain identity.

##### Inoculum Standardisation

**Figure 1:**
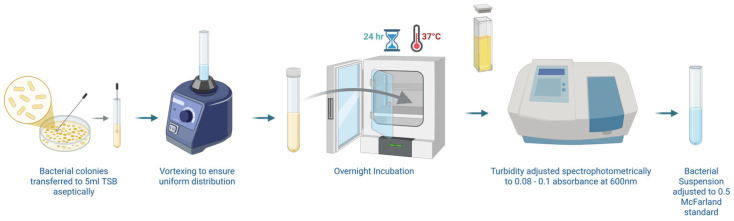
A 0.5 McFarland microbial inoculum preparation by the overnight growth method as recommended by CLSI guidelines. Created in BioRender. Sarwar, H. (2025) https://BioRender.com.

Overnight cultures were vortex-mixed; optical density was read at 600 nm and adjusted to 0.08–0.13 (≈1 × 10^8^ CFU mL^−1^, 0.5 McFarland standard) with sterile TSB. These suspensions were used immediately for all antimicrobial assays (Figure 1).

##### Zone of Inhibition Assay

The antibacterial activity of the F2l-BG45S5-T formulations loaded with vancomycin and teicoplanin was screened with an agar-well diffusion assay on tryptone soy agar (TSA). Plates were lawn-seeded with 100 µL of a 0.5 McFarland suspension of either MSSA (ATCC 6538) or MRSA (ATCC 43300) and allowed to dry for 5–10 min. Using a sterile 8 mm pipette tip, equidistant wells were cut in the agar, and each was charged with 50 µL of the test formulation solution (*n* = 3) at the prescribed concentration. Inoculated plates were incubated at 37 °C for 24 h, after which the diameters of the clear zones of inhibition surrounding each well were measured with a transparent millimeter ruler.

##### Time-Kill Assay

Time-kill kinetics of the vancomycin- and teicoplanin-loaded BG45S5 formulations were evaluated against MSSA (ATCC 6538) and MRSA (ATCC 43300). Overnight TSB cultures of each strain were diluted 1: 100 in fresh broth to give ~1 × 10^6^ CFU mL^−1^, and 3 mL aliquots were dispensed into sterile tubes. Drug-loaded Bioglass particles were added directly to a final concentration of 5 mg mL^−1^ (no pre-dissolution) and the suspensions were incubated at 37 °C with orbital shaking (225 rpm). At 0, 2, 4, 6, 8, 24, and 48 h, 100 µL was removed and serially ten-fold-diluted in PBS, and viable counts were performed in triplicate by the Miles and Misra drop plate method on TSA. Plates were incubated overnight (37 °C) and drops yielding 30–300 colonies were enumerated; results were expressed as CFU mL^−1^. All assays were run in triplicate with untreated cultures as growth controls.

#### 2.2.10. Cell Culture and Viability Assay

Saos-2 human osteosarcoma cells (ATCC HTB-85) were maintained in Dulbecco’s Modified Eagle Medium (DMEM; Gibco) supplemented with 10% (*v*/*v*) fetal bovine serum and 1% (*v*/*v*) penicillin/streptomycin (10,000 U mL^−1^). Cultures were incubated at 37 °C in 5% CO_2_ and passaged at 80–90% confluence. For sub-culturing, cells were rinsed with PBS, detached with 0.25% trypsin/EDTA (3–5 min, 37 °C), pelleted (1000 rpm, 5 min), resuspended in fresh medium, and counted with an automated hemocytometer.

Viability of Saos-2 cells exposed to BG45S5 formulations was evaluated by the MTT assay using conditioned-medium exposure, together with pure-compound controls (*n* = 3). Formulations were pre-incubated in complete DMEM at 5, 10 and 15 mg mL^−1^ for 24 h (37 °C, 250 rpm). Supernatants were filtered (sterile 0.20 µm PES), equilibrated for a further 24 h and applied to Saos-2 cultures at 1:1 (*v*/*v*) medium replacement for 24, 48, or 72 h.

Vancomycin HCl, teicoplanin, and trehalose dihydrate were freshly prepared in DMEM at 1000, 100, 80, 60, 40, and 20 µg mL^−1^ and tested under the same incubation periods.

After treatment, 20 µL MTT solution (5 mg mL^−1^ in PBS) was added per well and incubated for 4 h. Medium was removed, formazan was solubilised with 100 µL dimethyl-sulfoxide (DMSO), the plate was shaken (100 rpm, 10 min), and absorbance was read at 570 nm with a 630 nm reference. Cell viability was calculated as follows:

(4) $$Cell\;viability\;(\%)=\left(\frac{OD\;of\;treated\;cells-OD\;of\;blank}{OD\;of\;control\;cells-OD\;of\;blank}\right)\times 100$$

where OD is optical density. All conditions were tested in triplicate, and results were expressed as mean ± SD.

#### 2.2.11. Statistical Analysis

All statistical analyses were performed using GraphPad Prism version 10, Microsoft Excel, and custom Python—3.12 scripts. GraphPad Prism was used for conducting statistical tests, generating graphs, and calculating significance between treatment groups. Custom Python scripts were specifically employed for the analysis of in vitro drug release kinetics, including model fitting and evaluation of release parameters. Microsoft Excel was used for data organisation, basic calculations, and tabulation. All experimental data were expressed as mean ± standard deviation (SD), and appropriate statistical tests (e.g., one-way ANOVA or *t*-test) were applied where relevant, with *p*-values < 0.05 considered statistically significant.

## 3. Results

### 3.1. Particle Size Distribution by Laser Diffraction (LD) and Surface Topography by SEM of Blank and Antibiotic-Loaded Formulations

**Figure 2:**
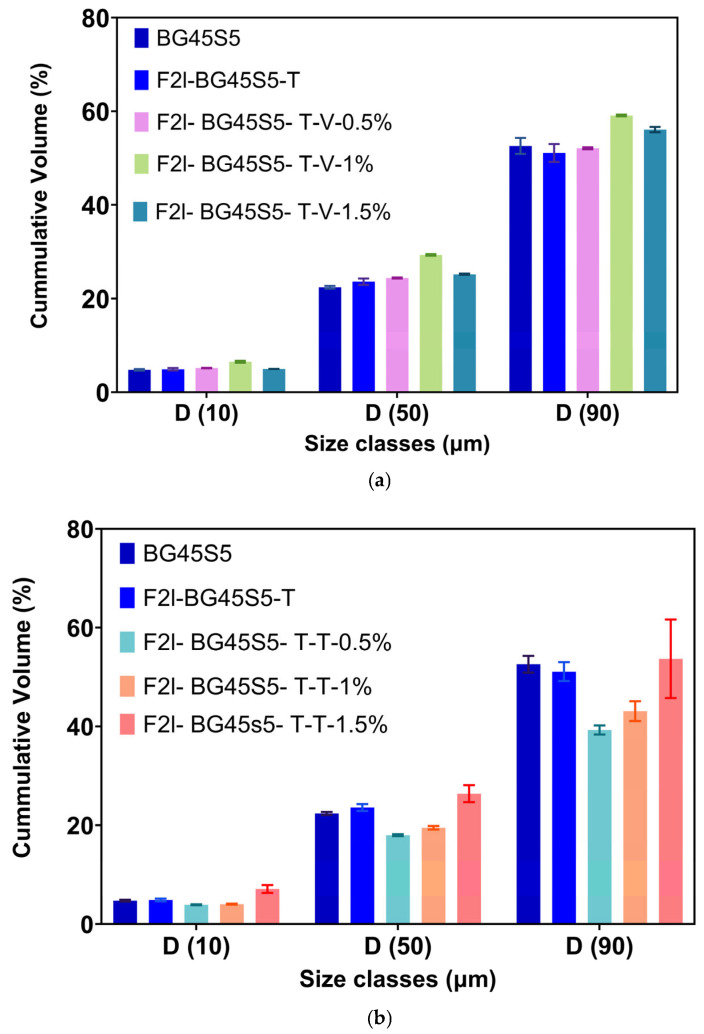
(**a**). Cumulative-volume particle-size classes (D_10_, D_50_ and D_90_) of BG45S5 core and its trehalose-coated analogue (F2l-BG45S5-T) before and after vancomycin loading (0.5, 1.0 and 1.5% *w*/*w*) *n* = 3. One-way ANOVA with Šídák correction was applied to column means. Only the 1.0% (*p* < 0.0001) and 1.5% (*p* < 0.001) formulations differed significantly from the BG45S5 core; all other pairwise comparisons were not significant (ns). (**b**). Cumulative-volume particle-size classes (D_10_, D_50_ and D_90_) of BG45S5 core and its trehalose-coated analogue (F2l-BG45S5-T) before and after teicoplanin loading (0.5, 1.0 and 1.5% *w*/*w*) *n* = 3. Relative to BG45S5, the 0.5% and 1% teicoplanin formulations showed significant decreases (*p* < 0.0001) and (*p* < 0.001), whereas the 1.5% formulation was not different (ns). The coated blank (F2l-BG45S5-T) did not differ from BG45S5 (ns). When compared with the coated blank, increases were again observed for the 0.5% and 1% formulations (*p* < 0.0001) and a modest decrease for the 1.5% formulation (*p* < 0.05).

**Figure 3:**
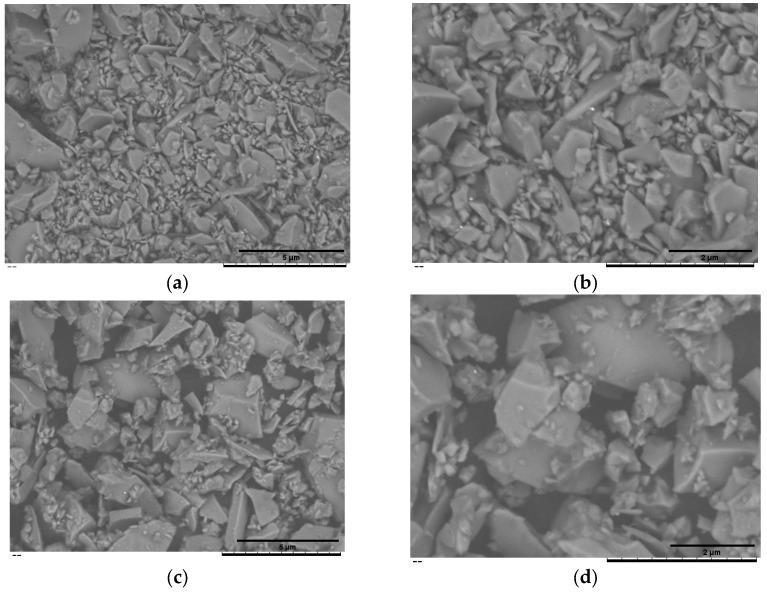
SEM of the uncoated BG45S5 core (**a**,**b**) and the trehalose-coated formulation F2l-BG45S5-T (**c**,**d**). Images were recorded at nominal magnifications of 1.5 k× (**a**,**c**) and 3 k× (**b**,**d**).

**Figure 4:**
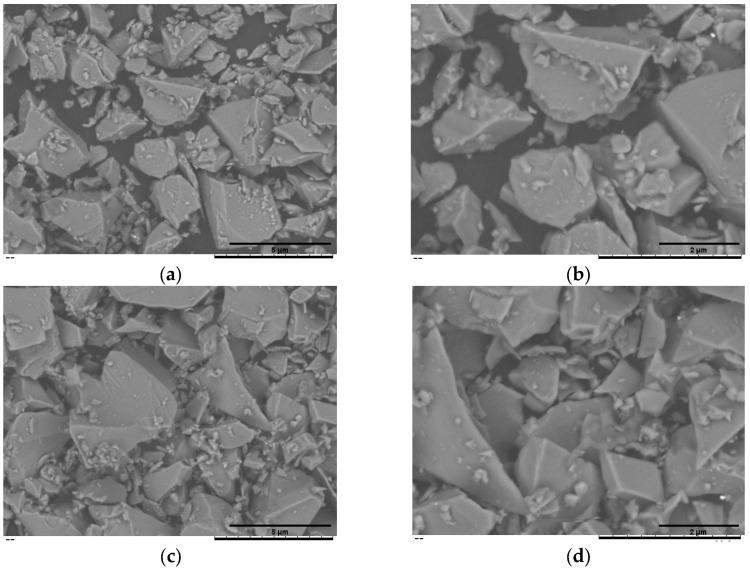

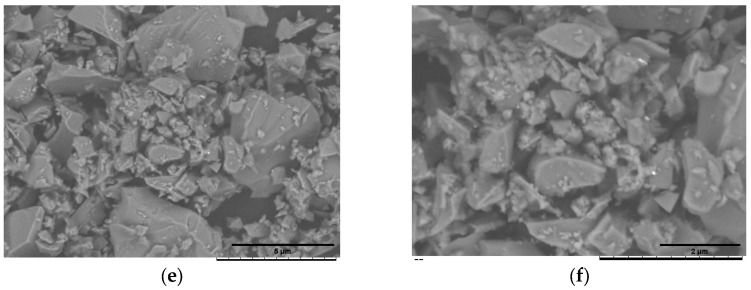
SEM of the vancomycin-loaded BG45S5 formulations. F2l-BG45S5-T-V-0.5% (**a**,**b**), F2l-BG45S5-T-V-1% (**c**,**d**), and F2l-BG45S5-T-V-1.5% (**e**,**f**). Images were recorded at nominal magnifications of 1.5 k× (**a**,**c**,**e**) and 3 k× (**b**,**d**,**f**).

**Figure 5:**
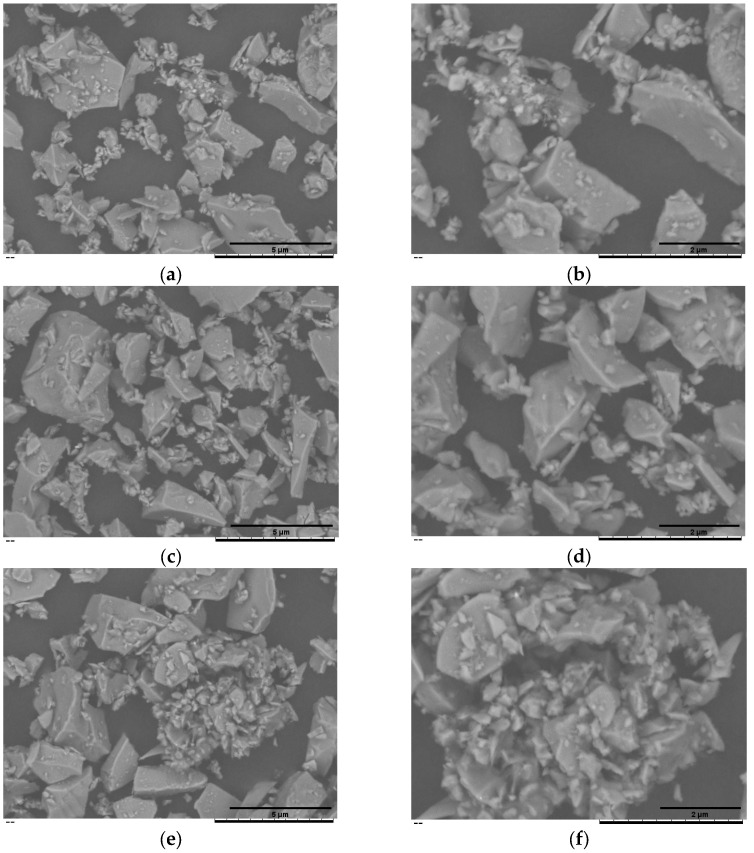
SEM of the teicoplanin-loaded BG45S5 formulations. F2l-BG45S5-T-T-0.5% (**a**,**b**), F2l-BG45S5-T-T-1% (**c**,**d**), and F2l-BG45S5-T-T-1.5% (**e**,**f**). Images were recorded at nominal magnifications of 1.5 k× (**a**,**c**,**e**) and 3 k× (**b**,**d**,**f**).

Figure 2a,b summarise the cumulative-volume classes (D_10_, D_50_ and D_90_) obtained for the uncoated BG45S5 core, the trehalose-coated blank (F2l-BG45S5-T), and the drug-loaded formulations. The trehalose layer alone produced only a marginal upward shift in D_50_. When 0.5% vancomycin was added, the distribution remained statistically indistinguishable from both BG45S5 and the coated blank. Loading at 1.0% enlarged D_50_ by ~5 µm and broadened the upper tail (significant change in D_90_), while 1.5% produced a smaller but still significant increase. D_10_ was unaffected in all cases, indicating that primary fines were not agglomerated by the coating or the drug. Teicoplanin-loaded formulations on the other hand were dose-dependent but opposite in direction. Incorporation of 0.5% and 1.0% teicoplanin reduced D_50_ by ≈6 µm and ≈4 µm, respectively, and narrowed the span between D_50_ and D_90_. The 1.5% loading restored the median size to the level of the trehalose blank and produced the broadest D_90_, suggesting partial aggregation or bridging at the highest concentration. These numerical trends are consistent with the surface morphology shown in the SEM micrograph, which confirms a smooth, consolidated surface morphology characteristic of non-porous, melt-quenched glass particles (Figure 3, Figure 4 and Figure 5).

### 3.2. Zeta Potential Analysis of the Blank and Drug-Loaded BG45S5 Formulations

**Figure 6:**
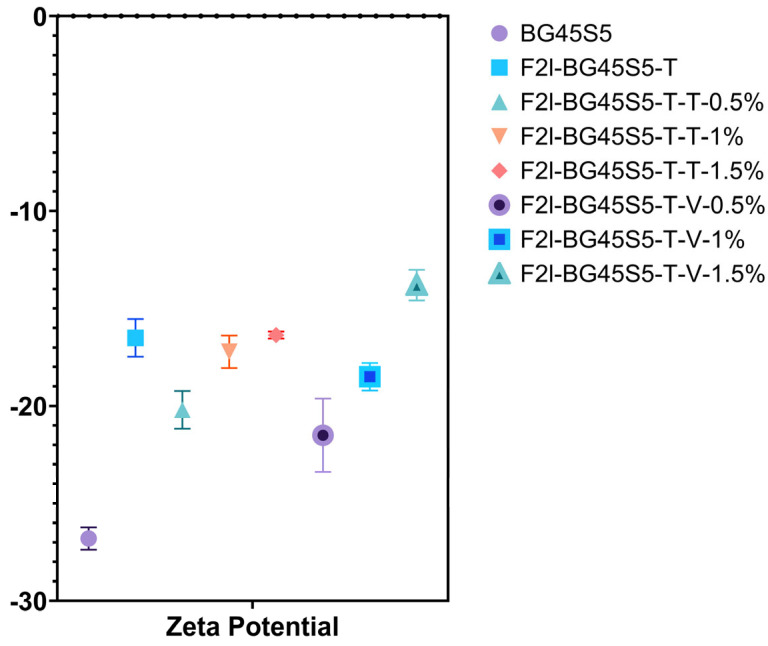
ZP(mV) of the cores, blank, and drug-loaded formulations (*n* = 5). The ANOVA analysis showed a significant increase in ZP in BG45S5 cores compared to coating with trehalose and drug loading (*p* < 0.0001).

Mean ZPs are plotted in Figure 6 (*n* = 5). Uncoated BG45S5 carried a strongly negative charge (−26.8 ± 0.577 mV). Trehalose adsorption lifted the value to −16.5 ± 0.971 mV, and every drug-loaded sample showed an upward shift relative to the uncoated BG45S5.

### 3.3. Drug-Loading Efficiency

**Figure 7:**
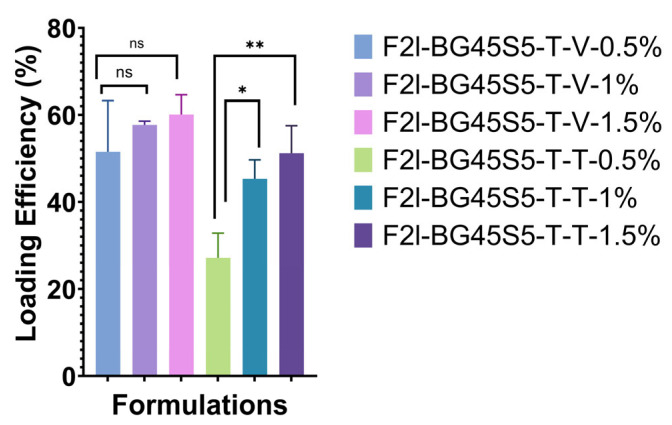
Loading efficiency of vancomycin-loaded (F2l-BG45S5-T-V) and teicoplanin-loaded (F2l-BG45S5-T-T) trehalose-primed Bioglass 45S5 at nominal doses of 0.5, 1.0, and 1.5 wt% (mean ± SD, *n* = 3). Vancomycin samples show no significant differences among doses (ns). Teicoplanin efficiency rises significantly from 0.5 wt% to 1.0 wt% (* *p* < 0.05) and further to 1.5 wt% (** *p* < 0.01); all teicoplanin values differ from their vancomycin counterparts at the same dose (not indicated for clarity; *p* < 0.05, Tukey post hoc test).

Drug-loading quantification (Figure 7) showed that the trehalose primer captured 52 ± 10%, 58 ± 3%, and 62 ± 4% of the vancomycin offered at 0.5, 1.0, and 1.5 wt%, respectively; one-way ANOVA revealed no significant difference among the three doses (*p* > 0.05). Teicoplanin displayed dose-dependent rises of 29 ± 6%, 46 ± 5% and 53 ± 7%with the jump from 0.5% *w*/*v* to 1.0% *w*/*v* significant (*p* < 0.05) and the increase to 1.5% *w*/*v* highly significant (* *p* < 0.01). At every nominal load, teicoplanin efficiencies were lower than vancomycin (Tukey post-hoc, *p* < 0.05), consistent with the lipophilic tail that limits adsorption onto the hydrophilic trehalose layer.

### 3.4. In Vitro pH Analysis of the Drug-Loaded Formulations in SBF

**Figure 8:**
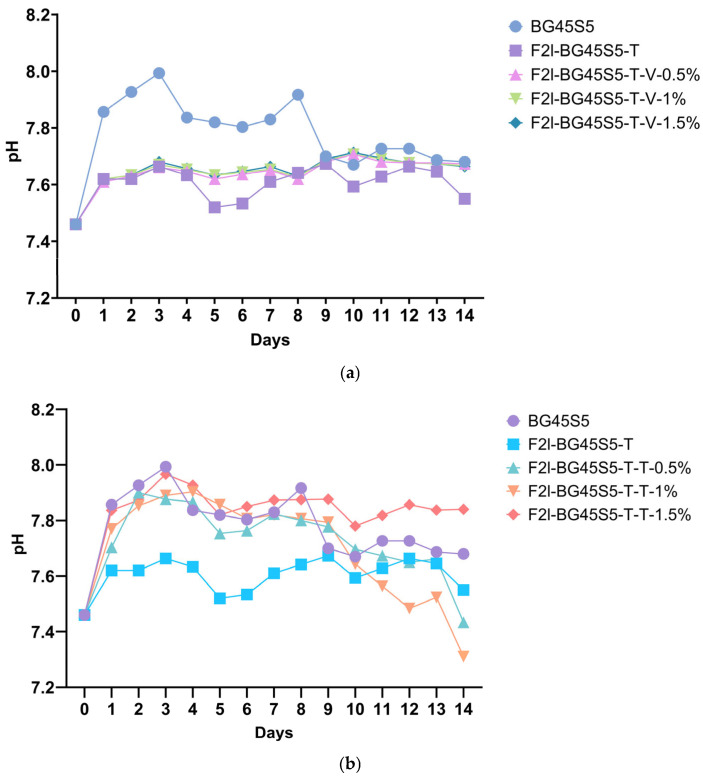
Time-dependent pH changes (SBF, initial pH 7.42) during 14 days’ immersion of BG45S5 and formulations (*n* = 3). (**a**) For vancomycin HCl-loaded F2l-BG45S5-T, the BG45S5 generated a significantly higher pH than all vancomycin-bearing samples (*p* < 0.0004) and the trehalose blank (*p* < 0.0001). (**b**) For teicoplanin-loaded F2l-BG45S5-T, the trehalose blank differed from BG45S5 (*p* < 0.01) and from the 1.5% load (*p* < 0.001); all other pairwise contrasts were not significant (ns).

The daily pH profiles of the uncoated glass, the trehalose blank, and the drug-loaded formulations are shown in Figure 8a,b. The alkaline excursion exhibited by the uncoated glass (BG45S5) was significantly greater than that of every vancomycin-containing sample, whereas differences among the coated blank and the three vancomycin loads were not significant. For teicoplanin, only the coated blank differed significantly from BG45S5 as well as from the 1.5% formulation; the remaining comparisons were non-significant. These statistics confirm that trehalose priming is the main factor moderating the pH rise, while antibiotic identity and dose exert a secondary, formulation-specific influence.

### 3.5. In Vitro Drug Release Profiling of Antibiotic-Loaded Bioglass Microparticles

**Figure 9:**
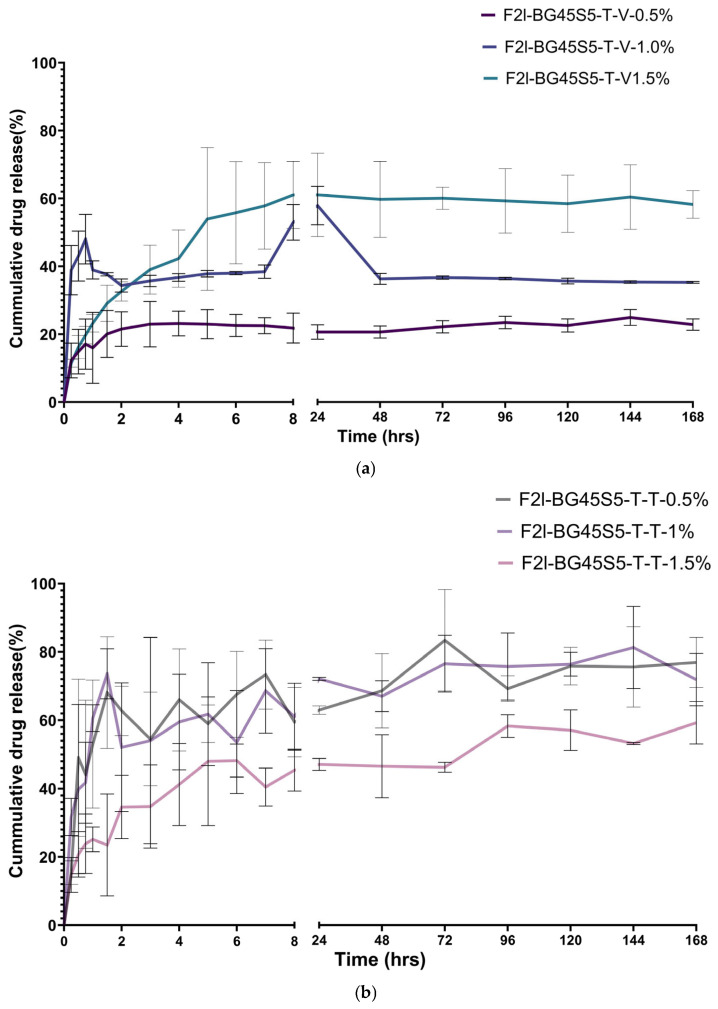
(**a**) Cumulative percentage drug release of BG45S5 formulations loaded with vancomycin HCl 0.5%, 1.0%, and 1.5% *w*/*v* over a period of 7 days. (**b**) Cumulative percentage drug release of BG45S5 formulations loaded with teicoplanin 0.5%, 1.0%, and 1.5% *w*/*v* over a period of 7 days.

**Figure 10:**
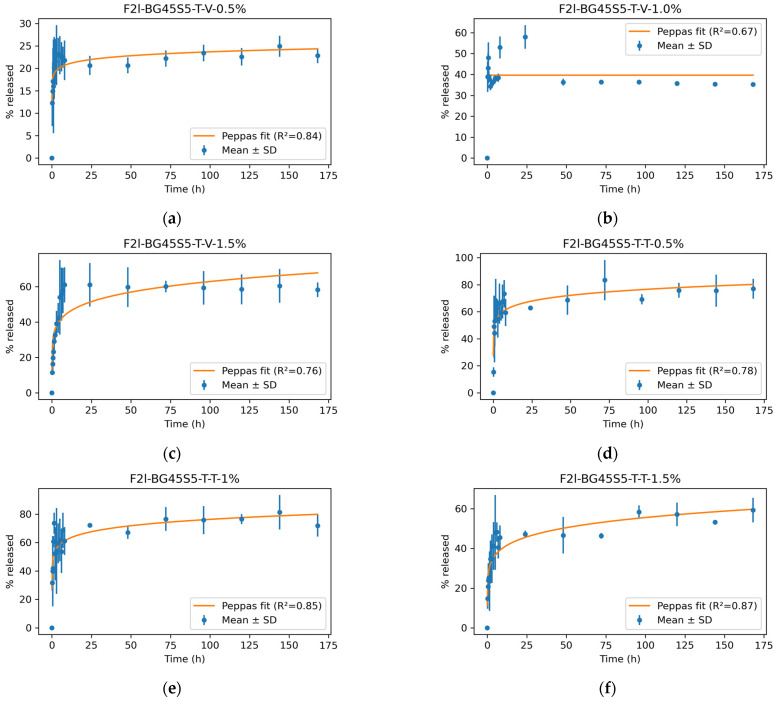
Pharmacokinetic model of release (goodness of fit—best fit) for formulations (**a**) F2l-BG45S5-T-V-0.5% (**b**) F2l-BG45S5-T-V-1% (**c**) F2l-BG45S5-T-V-1.5% (**d**) F2l-BG45S5-T-T-0.5% (**e**) F2l-BG45S5-T-T-1% and (**f**) F2l-BG45S5-T-T-1.5% showing R^2^ values respectively.

Mean release curves (*n* = 3) for teicoplanin (T-T) and vancomycin (T-V) are given in Figure 9a,b. All formulations displayed a biphasic pattern comprising (i) a burst phase during the first 2 h, followed by (ii) a slower, sustained phase that extended to 168 h. The kinetic models and the goodness of fit (R^2^) are represented in Figure 10.

### 3.6. Antimicrobial Assays

#### 3.6.1. Bacterial Strains and Molecular Confirmation

Two reference organisms were employed throughout the antibacterial work.

1. Methicillin-susceptible *Staphylococcus aureus* (MSSA)—ATCC 6538
2. Methicillin-resistant *Staphylococcus aureus* (MRSA)—ATCC 43300

To verify strain identity, the 16S-rDNA locus was amplified. PCR amplification with the universal primer pair 9 bfm/1512 uR generated a single amplicon of the expected length (~1.5 kb) from each culture. Sanger sequencing and BLASTn analysis returned 99.7% identity to the authentic reference sequences for ATCC 6538 and 99.5% to ATCC 43,300 (Table 3). No secondary peaks or indels were detected across the 1400 bp read length, confirming clonal purity.

#### 3.6.2. Zone of Inhibition Assay

**Figure 11:**
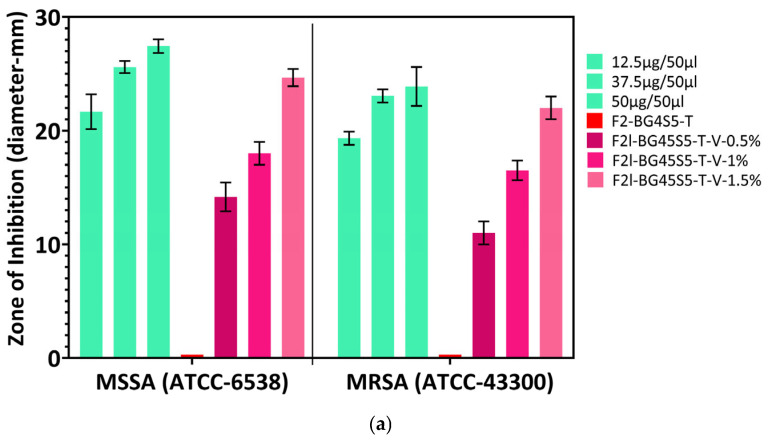

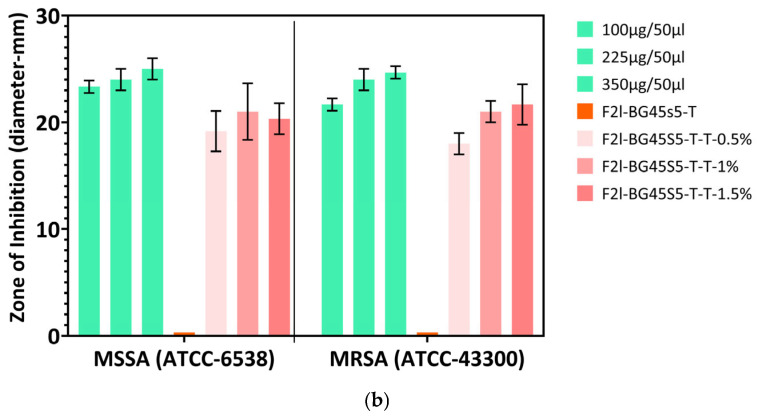
(**a**) Inhibition zones produced by glycopeptide-loaded Bioglass against methicillin-susceptible (MSSA ATCC 6538) and methicillin-resistant (MSRA ATCC 43300) strains (mean ± SD, *n* = 3) from vancomycin HCl-loaded formulations with positive control (free drug) and negative control (unloaded formulations). (**b**) Inhibition zones produced by glycopeptide-loaded Bioglass against methicillin-susceptible (MSSA ATCC 6538) and methicillin-resistant (MSRA ATCC 43300) strains (mean ± SD, *n* = 3) from teicoplanin loaded formulations along with positive and negative controls.

The agar-well diffusion test confirmed that antibacterial activity arose exclusively from the incorporated glycopeptides. The trehalose-coated blank (F2l-BG45S5-T) produced no measurable zone of inhibition (<1 mm), whereas the free drug controls formed wide, drug-limited clear zones (vancomycin 25–29 mm at 12.5–50 µg well^−1^; teicoplanin 22–27 mm at 100–250 µg well^−1^). When antibiotics were embedded in the Bioglass carrier, inhibition diameters increased systematically with loading. Against MSSA (ATCC 6538), the mean zone for vancomycin formulations grew from 15 ± 1 mm at 0.5% to 18 ± 1 mm at 1% and 25 ± 1 mm at 1.5% (Figure 11a). Teicoplanin displayed larger ZOIs at the same loads (≈20, 22 and 24 mm, respectively), reflecting its higher intrinsic potency. The MRSA (ATCC 43300) showed the expected reduction: zones were 1–3 mm smaller than the MSSA counterparts yet still exceeded the CLSI glycopeptide-susceptibility breakpoint (15 mm) at the 1% and 1.5% drug loading (Figure 11b). One-way ANOVA revealed a highly significant dose effect for each antibiotic (*p* < 0.001). Tukey’s test confirmed that every loaded sample differed from the blank (*p* < 0.0001) and that 1.5% > 1% > 0.5% for both strains (*p* < 0.01). Thus trehalose-primed Bioglass delivers glycopeptide concentrations sufficient to inhibit both MSSA and MRSA in a clear, dose-dependent fashion.

#### 3.6.3. Time-Kill Assay

**Figure 12:**
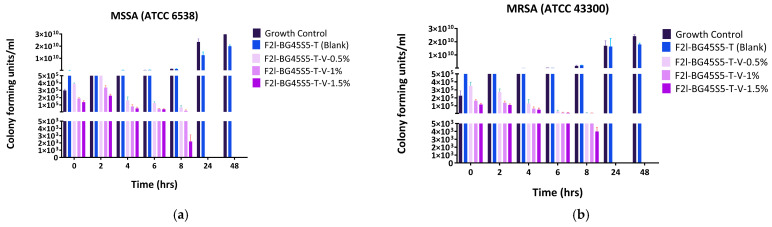

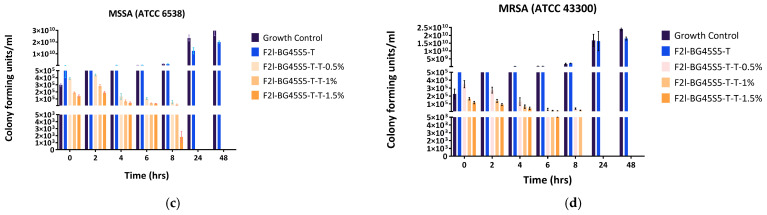
(**a**) Time-kill kinetics of glycopeptide-loaded Bioglass formulations against MSSA ATCC 6538 by vancomycin HCl-loaded BG45S5 formulations (mean ± SD, *n* = 3). (**b**) Time-kill kinetics of glycopeptide-loaded Bioglass formulations against MRSA ATCC 43,300 by vancomycin HCl-loaded BG45S5 formulations (mean ± SD, *n* = 3). (**c**) Time-kill kinetics of glycopeptide-loaded Bioglass formulations against MSSA ATCC 6538 by teicoplanin-loaded BG45S5 formulations (mean ± SD, *n* = 3). (**d**) Time-kill kinetics of glycopeptide-loaded Bioglass formulations against MRSA ATCC 43,300 by teicoplanin-loaded BG45S5 formulations (mean ± SD, *n* = 3).

Both antibiotics produced a rapid, load-dependent fall in viable counts, whereas the growth control and the trehalose blank (F2l-BG45S5-T) maintained logarithmic growth throughout the 48 h experiment (Figure 12a–d). Vancomycin-loaded formulations (F2l-BG45S5-T-V) showed clear dose dependence: 0.5% lowered MSSA counts by ≈1 log at 4 h and 2 logs at 8 h but never achieved the 3-log (99.9%) threshold; MRSA declined more slowly and remained ≥10^4^ CFU mL^−1^ at 24 h while 1% reached a 3-log kill at 6 h for MSSA and 8 h for MRSA, with no regrowth (≤10^3^ CFU mL^−1^) afterwards, whereas 1.5% achieved a 4-log reduction by 6 h in MSSA and by 8 h in MRSA; cultures were below the detection limit (10^2^ CFU mL^−1^) from 24 h onwards. On the other hand the teicoplanin-loaded formulations (F2l-BG45S5-T-T) acted faster, where 0.5% gave a 3-log drop in MSSA at 4–6 h and in MRSA at 8 h, but residual cells (10^3^–10^4^ CFU mL^−1^) persisted to 24 h, although 1% sterilised MSSA by 8 h and MRSA by 24 h while 1.5% sterilised both strains by 8 h with no detectable survivors thereafter.

### 3.7. Cell Viability Assay

**Figure 13:**
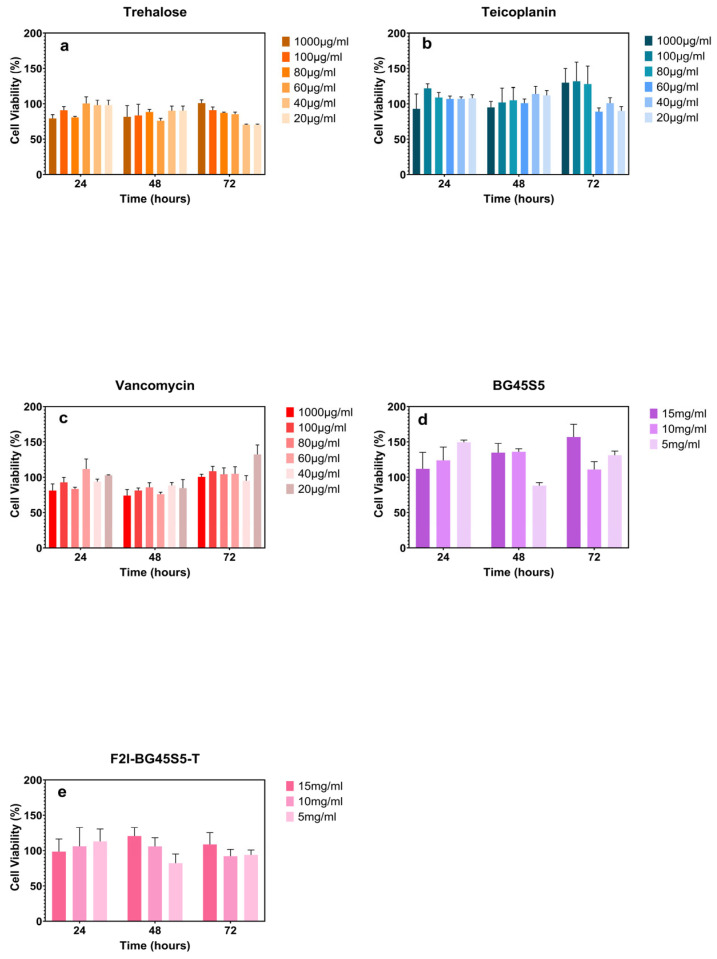
Saos-2 viability following 24, 48, and 72 h exposure to (**a**) trehalose (20–1000 µg mL^−1^), (**b**) teicoplanin (20–1000 µg mL^−1^), (**c**) vancomycin (20–1000 µg mL^−1^), (**d**) BG45S5 particles (5, 10, 15 mg mL^−1^), and (**e**) trehalose-coated BG45S5 particles (F2l-BG45S5-T) at matching doses. Data shows the mean ± SD (*n* = 3); >70% viability (ISO 10993-5 cytotoxicity threshold) [26]. No conditions differed significantly from the untreated control (one-way ANOVA, Bonferroni post hoc, *p* > 0.05).

Across all test samples, i.e., pure trehalose, free glycopeptides, uncoated BG45S5, and the test formulation F2l-BG45S5-T, Saos-2 viability remained ≥75% at every time point and concentration (Figure 13a–e).

## 4. Discussion

Trehalose physisorption is expected to add only a few nanometres of organic material to the Bioglass surface. As described in previous studies, physisorption refers to the reversible adsorption of molecules onto a substrate through weak van der Waals forces, hydrogen bonding, or electrostatic interactions; the multiple hydroxyl groups of trehalose readily form hydrogen bonds with hydrophilic surfaces, generating nothing more than an ultrathin organic shell [27]. Accordingly, the coated blank (F2l-BG45S5-T) retained virtually the same cumulative volume profile as the parent glass, suggesting the negligible size change. This suggests that trehalose, due to its higher glass transition temperature and superior water-binding capacity, forms a more uniform and stabilising matrix around the Bioglass core. The divergent behaviour of the two antibiotics can be rationalised in terms of molecular size and solid-state interactions: Vancomycin (MW ≈ 1.45 kDa) is amphiphilic and capable of forming an extended hydrogen-bond network with trehalose [28]. Teicoplanin (MW ≈ 1.9 kDa) contains a lipophilic tail that can intercalate between trehalose molecules, compacting the layer and drawing small particles together through hydrophobic interactions [29]. Overall, the trehalose primer layer provides a versatile platform that accommodates chemically distinct antibiotics, but the final particle-size characteristics are dictated by the specific drug–trehalose interaction regime. A recent example that illustrates how a trehalose primer layer is essentially size-neutral by itself yet can shift particle dimensions according to the particular cargo is described by Caballero-Florán et al. (2023) [30]. In another study Zhou et al. first photo ligated an ultrathin trehalose layer (~3 nm) onto mesoporous silica; the coating alone produced no significant size change. When isoniazid (small, hydrophilic) was loaded, the diameter remained within ±5 nm of the blank particles, whereas a bulkier hydrophobic analogue increased the Z-average by ~20 nm [31].

ZP (ζ) serves as a critical indicator of the colloidal stability and surface charge characteristics of particulate drug delivery systems. The stability of suspensions is directly influenced by the magnitude of the ZP, values between 0 and ±5 mV typically indicate rapid coagulation or flocculation, ±10 to ±30 mV suggests incipient instability, ±30 to ±40 mV correlates with moderate stability, ±40 to ±60 mV indicates good stability, while values above ±60 mV are considered to impart excellent stability to the dispersion [32]. Beyond its role in physical stability, the ZP also has important biological implications [33]. Negatively charged surfaces have been shown to enhance the attachment and proliferation of osteoblast-like cells, making them particularly suitable for orthopedic applications [34,35]. Secondly, the reduced electrostatic repulsion between the particle and the negatively charged bacterial envelope means that the released antibiotic is located at the interface, enhancing bactericidal efficiency. Elahpour et al. demonstrated this with cation-adjusted bioactive glasses: doping 45S5 with Cu^2+^ and Zn^2+^ shifted the surface ζ-potential from −26 mV to +4 mV and halved the minimum contact time required to obtain a 3-log reduction of planktonic SA, while undoped glass under identical ion-release conditions produced only a 1-log drop [36]. In the current study, the BG45S5 surface is dominated by deprotonated silanols, giving a ZP close to −27 mV, while after being loaded with drug the ZP values gives an upward shift, comparable to values reported for 45S5 powders and temporal variations [37].

In this study we aimed to investigate the pH stability of various BG45S5 formulations in SBF at 37 °C over a period of 14 days. BG45S5 elevates extracellular pH through rapid Na^+^/Ca^2+^–H^+^ exchange and silicate network dissolution [38]. The spike to >7.9 observed for the bare core agrees with classical SBF studies reporting increases of 0.4–0.6 units in the first 48 h [39]. Importantly, all antibiotic-bearing formulations preserved a pH window (7.4–7.8) conducive to osteoblast proliferation; values above 8.0 have been linked to decreased cell viability on bioactive substrates [40]. Thus, the trehalose primer not only moderates the early alkaline burst but, when combined with appropriate antibiotic loading, keeps the micro-environment within the optimal physiological range while the glass continues to form hydroxycarbonate apatite; a balance that favours simultaneous antibacterial action and bone regeneration.

For an antibacterial bone graft, how an antibiotic is released is as critical as how much it is released. Implant-associated infections are seeded within the first few hours after surgery, yet mature SA biofilms can tolerate antibiotic concentrations >1000-fold above the planktonic minimum inhibitory concentration (MIC) [41]. An ideal carrier therefore delivers (i) a rapid, bactericidal “hit” that prevents early adhesion, followed by (ii) a sustained, sub-cytotoxic flux that suppresses any residual bacteria until vascularised tissue can clear the wound [42]. The present Bioglass–trehalose platform was designed with precisely that dual objective, and the release data confirm that both antibiotics can be tuned into the desired pharmacodynamic window. Trehalose dissolves within minutes, carrying with it surface bound drug and creating the burst phase whose magnitude scales with loading (Figure 9). Once the primer layer is gone, water permeates the 45S5 network and release becomes diffusion controlled. However, curve-fitting of our mean profiles showed that all six formulations were better described by the Korsmeyer–Peppas model (R^2^ = 0.67–0.87) than by Higuchi, with diffusion exponents *n* = 0.19–0.26 indicating Fickian release through the trehalose-primed glass matrix. This confirms that, after the initial trehalose-mediated burst, drug transport is governed by concentration-driven diffusion from the Bioglass network [43,44]. The total maximum release of vancomycin, ~60%, was observed from the highest concentration loading, which is less than the previously reported release for vancomycin from poly(3-hydroxybutyrate-co-3-hydroxyvalerate) (PHBV)-coated BG45S5 [45]. Only teicoplanin at 1.5% deviates (*n* ≈ 0.6), reflecting partial pore occlusion by its lipophilic tail. A parallel effect showed that increasing teicoplanin loading in PLGA microspheres from 1.0% to 1.5% created a 15–25 nm surface “skin,” lowered surface porosity by 38%, and shifted the release exponent from 0.45 to 0.59 [25]. Thus, our elevated *n* value likely arises from the same tail induced occlusion mechanism. Vancomycin, lacking such a tail, does not obstruct the pores; even at 1.5% it retains Fickian release. The total cumulative release of teicoplanin with highest drug loading concentration was found to be almost 60%, which is more than the previously reported studies for 7 days. This is markedly higher than the 34–40% reported by Huang et al. for nanoporous bioactive-glass granules loaded with the same drug (30 µg mg^−1^) and tested under identical SBF conditions for one week. Huang’s SEM images revealed partial pore blocking and a residual drug reservoir on day 7, which explains their lower plateau. Our greater extent of release therefore indicates that the trehalose primer minimises tail-induced occlusion, allowing a larger fraction of teicoplanin to diffuse out while still preserving the burst/sustain profile for 7 days and longer [46].

Genetic confirmation eliminates two common confounders in antimicrobial research: phenotypic drift after repeated subculture and accidental strain substitution. The ≥99% 16S-rDNA homology obtained here ensures that the organisms used throughout the zone-of inhibition and time kill assays are identical to the internationally accepted quality control strains [47]. The free-drug wells define the maximal ZOIs attainable by unobstructed diffusion in agar; loaded particles necessarily yield smaller zones because the antibiotic must first desorb or diffuse from the carrier, a phenomenon classically demonstrated for vancomycin-PMMA beads, whose inhibition diameter was 40% narrower than that of an equal mass of free drug [48]. Nevertheless, diameters ≥20 mm at 1% and ≥ 24 mm at 1.5% are well above the CLSI glycopeptide-susceptibility breakpoint of 15 mm for SA [49,50], indicating clinically relevant activity. Teicoplanin gave larger inhibition zones than vancomycin at the same loading, consistent with its greater intrinsic potency and slightly faster burst release [22]. The ≈ 10% reduction observed for MRSA relative to MSSA mirrors the two-fold higher glycopeptide MIC typically reported for resistant strains [51,52], but this difference is fully overcome at the 1.5% drug-loaded formulations, showing that the Bioglass carrier can still deliver sufficient local concentration. Finally, the ZOI data reinforce the release–kill relationship as shown in Figure 9 and Figure 10; the trehalose controlled burst supplies bactericidal levels rapidly enough to inhibit lawn growth, while simple dose escalation provides a lever to match potency with pathogen susceptibility.

The time-kill results mirror the physicochemical trends observed earlier and thus strengthen the overall mechanistic picture of the trehalose-primed Bioglass system. Formulations that released ≥70% of their payload within the first 3 h, teicoplanin at 1.0 and 1.5% and vancomycin at 1.5%, were precisely the ones that achieved a ≥ 3-log reduction in planktonic counts within 6–8 h (Figure 12). By contrast, the slower releasing 0.5% batches never sterilised the cultures before 24 h. This temporal concordance confirms that the trehalose-controlled burst is the primary driver of rapid kill, just as previously reported for glycopeptide-loaded 45S5 scaffolds [53,54]. Zone-of-inhibition diameters grew in the same order—0.5 < 1.0 < 1.5%—and the 24–25 mm ZOIs produced by the highest loads correspond to soluble concentrations that exceed the MIC by two orders of magnitude as shown in Figure 9. The fact that these large diameters translate into complete sterilisation in TKA indicates that diffusion from the particles is fast enough to recreate agar level concentrations in liquid culture, provided the burst fraction is high enough. All antibiotic-bearing formulations kept the SBF pH between 7.4 and 7.8 (Figure 8). This mild alkalinity neither inactivates glycopeptides nor harms osteoblasts, but it does impair SA biofilm formation; studies have shown a 30–40% reduction in adhesion when the local pH rises above 7.7 [55]. The modest pH increase therefore acts synergistically with the antibiotic: it slows initial colonisation while the burst supplies bactericidal levels, explaining why even MRSA is eradicated once the 99.9% threshold is passed. Teicoplanin’s larger agar ZOIs and shorter kill times, despite a slightly lower cumulative release after 24 h, reflect its lower MIC (0.5–1 µg mL^−1^ vs. 1–2 µg mL^−1^ for vancomycin) and higher membrane affinity [56,57]. Vancomycin compensates only when the drug load is increased to 1.5%, underscoring the value of the Bioglass matrix, which tolerates this higher payload without pore blocking (Fickian *n* ≈ 0.49).

The MTT results confirm that every individual building block of the formulations, trehalose, BG45S5, and either glycopeptide, falls within the ≥70% viability threshold commonly accepted for biomaterials (ISO 10993-5) [58]. The absence of a dose response up to 15 mg mL^−1^ for glass particles is consistent with earlier reports that BG45S5 supports osteoblast proliferation provided the medium pH is kept below 8.0; our pH data (Figure 8) remained within 7.8, which likely explains the maintained viability. Likewise, the high tolerability of vancomycin and teicoplanin at 1000 µg mL^−1^ aligns with their routine systemic use in bone-infection therapy. These findings, coupled with the strong antibacterial performance, indicate that the trehalose-primed bioactive-glass system offers an excellent therapeutic window: bactericidal for pathogens yet non-toxic for osteoblastic cells.

## 5. Conclusions

When bone integrity is compromised, pathogens can establish themselves more rapidly than the tissue can regenerate. The trehalose-primed BG45S5 platform in this study counters that race on three fronts: it preserves the parent glass’s favourable particle size, moderates the early alkaline surge, and delivers a tunable “burst-plus-sustain” release of the antibiotic, vancomycin or teicoplanin, to the site of infection. The primer layer adds only nanometres of organic coating yet dictates drug–glass interactions: vancomycin produces a thin, hydrogen-bonded over shell, whereas teicoplanin’s lipophilic tail compacts the film. These molecular differences translate directly into colloidal charge, release kinetics, and, ultimately, antibacterial efficacy. The antibacterial efficacy of trehalose-primed Bioglass 45S5 is dictated by a multifactor interplay: (i) drug-loading efficiency—higher encapsulation secures a larger reservoir for release; (ii) burst/sustained release profile—the initial burst (>70% within 3 h) determines how quickly planktonic bacteria are sterilised, whereas Higuchi-type diffusion maintains supra-MIC levels for several days; (iii) surface charge (ζ-potential)—moderately negative values (−15 to −20 mV) minimise electrostatic repulsion with the bacterial envelope and localise the antibiotic at the particle interface; (iv) pH modulation—the trehalose layer tempers the 45S5 alkaline surge, keeping the micro-environment at pH 7.4–7.8, a range that both preserves glycopeptide activity and inhibits early biofilm formation; (v) antibiotic physicochemistry—vancomycin’s amphiphilicity versus teicoplanin’s hydrophobic tail influences pore occlusion, release exponent (*n*) and ultimately kill kinetics; (vi) particle size/surface area—sub-90 µm D_90_ ensures injectability while providing sufficient surface for rapid ion and drug exchange. All antibiotic-bearing formulations retained ≥70% Saos-2 viability at doses up to 15 mg mL^−1^ and exceeded the CLSI breakpoint in agar (≥20 mm at 1%, ≥24 mm at 1.5%). Time-kill assays confirmed the mechanistic link: batches that released ≥70% of their payload in the first three hours achieved ≥3-log reductions in both MSSA and MRSA within eight hours, with no regrowth to 48 h. Thus, a 1% drug load is sufficient for methicillin-susceptible infections, while 1.5% overcomes MRSA’s higher MIC without compromising cell compatibility. The in vitro data obtained in this work position trehalose-primed Bioglass as a promising dual-function drug delivery option that is osteoconductive and locally bactericidal. A detailed FT-IR/ss-NMR study of trehalose–antibiotic interactions on 45S5 is underway and will be reported separately. In vivo studies should be undertaken to expedite these materials into clinical applications.

## Figures and Tables

**Table 2:** Chromatographic conditions for the quantitative detection of vancomycin HCl and teicoplanin from the in vitro drug release samples.

Parameter	Vancomycin HCl	Teicoplanin
Instrument	Shimadzu (Shimadzu Corporation, Kyoto, Japan) LC-20AT pump, SIL-20A autosampler, SPD-M20A UV detector; software: LC LabSolutions (Version: 1.23 SP1)	Shimadzu LC-20AT pump, SIL-20A autosampler, SPD-M20A UV detector; software: LC LabSolutions
Column/guard	Phenomenex Luna^®^ C18(2) (150 × 4.6 mm, 5 µm) + Phenomenex K10-4282 guard	Phenomenex Luna^®^ C18(2) (150 × 4.6 mm, 5 µm) + Phenomenex K10-4282 guard
Mobile phase (*v*/*v*)	Ultrapure water: Methanol: Acetonitrile (80:15:5) + 0.05 M Orthophosphoric acid	Acetonitrile: ultrapure H_2_O (90:10)
Flow rate	0.9 mL min^−1^	0.9 mL min^−1^
Detection wavelength	280 nm	280 nm
Injection volume	100 µL	50 µL
Retention time (≈)	5.5 min	2 min

**Table 3:** 16S rDNA strain confirmation.

Isolate	Best GenBank Hit (16S rRNA)	Identity (%)	Query Cover (%)	Accession
MSSA ATCC 6538	*Staphylococcus aureus* strain ATCC 6538	99.7	100	MT573388.1
MRSA ATCC 43300	*Staphylococcus aureus* strain ATCC 43300	99.5	100	MW464217.1

## Data Availability

All data generated or analyzed during this study are included in this article.

## Abbreviations

The following abbreviations are used in this manuscript:

ANOVA	Analysis of variance
ATCC	American Type Culture Collection
BG45S5	Bioglass 45S5
CFU	Colony-forming units
D	Diameter
DMSO	Dimethyl sulfoxide
EDTA	Ethylenediaminetetraacetic acid
HPLC	High pressure liquid chromatography
MRSA	Methicillin resistant staphylococcus aureus
MSSA	Methicillin susceptible staphylococcus aureus
MTT	3-(4,5-dimethylthiazol-2-yl)-2,5-diphenyltetrazolium bromide
PMMA	Polymethylmethacrylate
RSD	Relative standard deviation
SA	Staphylococcus aureus
SBF	Simulated body fluid
SD	Standard deviation
TKA	Time-kill assay
ZOI	Zone of inhibition

## Appendix A

This appendix describes the scheme of formulations used for optimisation.

**Table A1:** Coded formulations before optimisation.

Ratio of Bioglass/Carbohydrate (*w*/*w*)	45S5: Sucrose	45S5: Trehalose
1:0.25	F1a-BG45S5-S	F2a-BG45S5-T
1:0.5	F1b-BG45S5-S	F2b-BG45S5-T
1:0.75	F1c-BG45S5-S	F2c-BG45S5-T
1:1	F1d-BG45S5-S	F2d-BG45S5-T
1:1.25	F1e-BG45S5-S	F2e-BG45S5-T
1:1.5	F1f-BG45S5-S	F2f-BG45S5-T
1.1.75	F1g-BG45S5-S	F2g-BG45S5-T
1:2	F1h-BG45S5-S	F2h-BG45S5-T
1:2.25	F1i-BG45S5-S	F2i-BG45S5-T
1:2.5	F1j-BG45S5-S	F2j-BG45S5-T
1:2.75	F1k-BG45S5-S	F2k-BG45S5-T
1:3	F1l-BG45S5-S	F2l-BG45S5-T
